# Pediatric Thyroid Carcinoma Incidence and Temporal Trends in the USA (1973–2007): Race or Shifting Diagnostic Paradigm?

**DOI:** 10.5402/2012/906197

**Published:** 2012-03-06

**Authors:** Laurens Holmes, Jobayer Hossain, Franklin Opara

**Affiliations:** ^1^American Health Research Institute, 427 West 20th Street, Suite 707, Houston, TX 77008, USA; ^2^Nemours Biomedical Research, Nemours Center for Childhood Cancer Research, 1700 Rockland Road, Wilmington, DE 19803, USA; ^3^Department of Biological Sciences, University of Delaware, 118 Wolf Hall, Newark, DE 19716, USA; ^4^Spatial Epidemiology Division, American Health Research Institute, 427 West 20th Street, Suite 707, Houston, TX 77008, USA

## Abstract

Pediatric thyroid carcinoma is relatively uncommon. But variability in incidence rate by race, sex, age at onset/diagnosis, and geographic local had been observed in adult thyroid carcinoma in the USA. We aimed to examine the patterns, rates, and temporal trends of thyroid carcinoma among pediatric patients (0–19 years) between 1973 and 2007. The Surveillance, Epidemiology, and End Results (SEER) data of the National Cancer Institute were utilized. Data were available on sex, age at diagnosis, race/ethnicity, and geographic locale (9 SEER registries) and were used for rates and trends computation. The frequency and percentage, percent changes (PCs) were calculated by using 1 year of each endpoint. Similarly, the annual percent changes (APCs) were calculated as well, with APCs estimated using weighted least square methods. Between 1973 and 2007, 1,360 thyroid cancer cases were ascertained in the 9 SEER areas (*n* = 247,638,734) in the USA. The percent change was 47.9, while the APC was significantly different from 0, 1.0 (95% CI: 0.5–1.6, *P* < 0.0001). The rate ratio (RR) was significantly lower in 1975 (RR: 0.62, 95% CI: 0.38–0.98, *P* = 0.03) relative to the rate between 1973 and 2007 (RR: 1.60, per 100,000, 95% CI: 1.50–1.70), but higher in 2007 (RR: 2.3 per 100,000, 95% CI: 1.70–3.10; RR: 1.44, 95% CI: 1.05–1.93, *P* = 0.02). The rate was significantly higher in whites relative to blacks, highest among age group of 15–19 years and girls, and in some SEER registries, with some significant PC in Connecticut. This temporal trend study of pediatric thyroid carcinoma indicates increase in the rate of this malignancy given the percent change and the annual percent change between 1973 and 2007. In addition, the incidence was higher among girls, lower among blacks, highest in age group of 15–19 years, and relatively higher in SEER registries with predominantly white or Hispanic populations.

## 1. Introduction

 Thyroid carcinomas are tumors that originate from epithelial cells and are relatively rare. Whereas the follicular and papillary, which are highly differentiated cells and anaplastic (poorly differentiated) types, arise from follicular cells, medullary cells types arise from parafollicular C cells, which are cells associated with calcitonin production [1]. The prognosis for papillary and follicular carcinoma subtypes had been reported to be good, while the anaplastic carcinoma is poor in prognosis. The papillary and follicular cell types are the most common, accounting for an estimated 80–90% of all thyroid carcinomas. Relative to leukemia, CNS/brain tumor, and lymphoma in children, thyroid cancer is rare, affecting approximately 1 in every 1000 to 2000 children in the United States. Based on the Surveillance Epidemiology and End Results (SEER) Cancer Registries, the age-standardized incidence rates (1987–1991) were 2.5 per 100,000 for males and 6.4 per 100,000 for females [2]. Sex and racial variability has been observed in SEER registries. The incidence from the SEER (1983–87) was 1.0 in black males and 2.2 in white males per 100,000, while the incidence was 2.7 for black females and 5.8 per 100,000 for white females [3]. Sex variability as shown in nonmedullary differentiated cell types peaks after puberty and increases to three-fold differences during child-bearing years in women and declining thereafter.

 In general, the incidence is two times as likely in whites compared to blacks, with Asians, compared to white or blacks, more likely to be diagnosed with thyroid cancer [4]. With respect to presentation, there is variability in tumor subtype by sex, with female-to-male ratio of 1 : 3 for papillary and follicular cell types, but no such female-male ratio is seen in medullary carcinoma, which may be due to the implication of increased estrogen receptors in papillary and follicular cell types [5]. 

 Time trends in papillary and follicular thyroid cancer indicated an increase until about 1980 [6]. Whereas, in the USA, incidence increased between 1947 and 1978, rates tended to plateau between 1978 and 1979 [7]. 

 Like in most pediatric malignancy, the risk factors for pediatric thyroid cancer are not well understood. This may be due to apparent restricted or limited epidemiologic interest in this rare but relatively good prognosis and survival tumor, as well as the several histopathological subtypes and prognostic variability associated with the cell subtypes. Epidemiologic data strongly suggest ionizing radiation, which may tend to increase the incidence in children, given extensive use of radiation today in the treatment of other childhood malignancies (leukemia, lymphoma, CNS/brain, Wilms' tumor, neuroblastoma, etc.). Inherited susceptibility is seen an estimated 3% of thyroid cancer, with such presentations suggested to be clinically more aggressive [8]. Germline mutations in the ret protooncogene, which is an autosomal dominant inheritance with high penetrance and variable expression have been associated with an estimated 20% of medullary thyroid cancer. Familial contributions to thyroid cancer had been observed in which the rate for the offspring of thyroid cancer patients increased by multiple-folds [9]. Evidence is conflicting on specific gene alteration implicated in thyroid cancer. However, growth factor receptors, oncogenes (ras gene, ret protooncogene, and ret/ptc), and tumor-suppressor genes (p53 point mutation) had been associated with thyroid carcinogenesis [10, 11]. The implication of the growth factor in thyroid carcinogenesis is plausible given that follicular cell growth is regulated by growth factors while thyroid cell proliferation is essentially controlled by thyroid-stimulating hormone.

 Epidemiologic predisposing and protective factors not established in thyroid carcinogenesis include smoking, diet (high iodine level and increase risk, vegetables and decreased risk), alcohol (increased risk), hormones (elevated TSH secretion, puberty, pregnancy, and labor, oral contraceptive, thyroidectomy, goitrogens, and radiation therapy), weight (increased BMI in women and increasing risk), ionizing radiation (established risk factor), with doses as low as 0.1 Sievert associated with increased risk, and is dependent on the age at exposure—more sensitive during early years [12], and diseases and therapeutics (hyper- or hypothyroidism, thyroid adenoma, goiter, thyroiditis, iodine deficiency/endemic goiter, and breast cancer) [13]. These risk and possible predisposing factors remain inconclusive except ionizing radiation, due probably to confounding effects, such as the association between breast cancer and thyroid carcinoma, diets, and alcohol.

 Epidemiologic risk factors in childhood or pediatric thyroid carcinoma have not been explored relative to the studies in adults. However, hormones, somatic events, inherited susceptibility, and exposure to ionizing radiation are factors that are similarly implicated in pediatric thyroid carcinoma. Additionally, data suggest ionizing radiation with increasing predisposition to pediatric thyroid cancer. Children treated with external X-irradiation and by external exposure to active iodine for enlarged thymus glands were demonstrated to develop papillary-follicular thyroid carcinoma after a latency of an estimated ten years [14]. Epidemiologic data on temporal incidence trends are needed on pediatric thyroid carcinoma that will allow us to have comparable data as in adult presentation with respect to sex variability, racial/ethnic variance, geographic locale, and age at onset in the United States.

 The present study sought to examine the incidence trends (percent change and annual percent change) in pediatric thyroid cancer (1973–2007) using the Surveillance Epidemiology and End Results (SEER) dataset from the 9 SEER registries and determine the incidence by sex, race, age and onset, and geographic locale.

## 2. Materials and Methods

After an approval from the relevant IRB, we conducted a temporal trend study to assess the incidence rate/trend in pediatric thyroid cancer as well as to examine the incidence rate/trend by race, sex, age group at diagnosis, and SEER registries (geographic locale).

### 2.1. Study Population

The study population comprised children of age 0–19 years with newly diagnosed thyroid cancer between 1973 and 2007.

### 2.2. Variables

This study assessed the incidence rate/trend in the overall pediatric population at risk and examined the rate differences by sex, race, age at diagnosis, and SEER geographic areas. Sex was classified as male and female for boys and girls, respectively. Race was classified as black, white, and others, where others comprised American Indian-Alaska native, Asia-Pacific Islander, and others. The age at diagnosis was grouped into (a) 0–4 years, (b) 5–9 years, (c) 10–14 years, and (d) 15–19 years. The SEER registries represent geographic locale where the tumors were diagnosed along with the pediatric population at risk. The SEER areas used in this study were the seven initial registries, which began in 1973, plus Seattle and Atlanta Metropolitan registries, which started in 1975.

### 2.3. Data Source

The National Cancer Institute (NCI), an agency of the National Institute of Health (NIH), supports the Surveillance Epidemiology and End Results (SEER) program. SEER collects data on all malignancies diagnosed and confirmed through histopathology (biopsy) in all SEER eligible cancer registries. The initial registries comprised San Francisco (SA), Connecticut (CN), Detroit Metropolitan (DM), Hawaii (HI), Iowa (IO), New Mexico (NM), Seattle (SE), Utah (UT), and Atlanta Metropolitan (AM). Of these nine registries SE and AM joined SEER in 1975 implying that thyroid cancer incidence data were not available for these two registries during 1973 and 1974. These 9 SEER registries represent an estimated 11% of the US population.

SEER data had been validated and shown to be reliable in population-based studies. SEER employs highly qualified data processor and entry clerks which have resulted in the high quality of the SEER data for research purposes. While SEER remains a reliable source for cancer incidence and mortality, estimate of survival using these data remains questionable due to the lack of variables on treatment such as chemotherapy as well as variable survival times.

### 2.4. Statistical Analysis

We obtained data on the thyroid cancer counts as well as the pediatric population at risk in the nine SEER registries. The nine oldest SEER area data from 1973 to 2007 were used to calculate the age-adjusted rates for the geographic locale, race, sex, and age at diagnosis (0–19) Years. Rates, standard errors and 95% Confidence Intervals for the rates were calculated. The rates were per 100,000 and age-adjusted to the 2000 US standard population. Percent change was calculated using 1 year for each endpoint, while the annual percent change (APC) was calculated using weighted least squares method. The APC is the average rate of change in a rate over several years and is used to measure trends over time (1973–2007). Statistical significance (meaning APC was significantly different from zero) was assessed, utilizing *P* < 0.05 for APC and 95% confidence interval (CI) for rates and trends. All statistical analyses were performed using the most recent SEER statistical package, SEER*Stat 6.6.2.

## 3. Results

This result presents the study of pediatric thyroid cancer that assessed long-term temporal trends in this malignancy. The data comprised all pediatric thyroid carcinoma diagnosed between 1973 and 2007. The diagnosis and the population at risk represent 9 SEER registries with an estimated 11% of the total US population in terms of children, 0–19 years of age. During this period, there were 1,360 thyroid carcinomas diagnosed, which consisted of all histological subtypes (papillary, follicular, medullary, and anaplastic).

Table 1 demonstrates the annual incidence of pediatric thyroid carcinoma in 1973–2007 in the SEER registries. The incidence rate ranged from 0.4 to 0.7 per 100,000 children of 0–19 years. The percent change was 47.9, while the APC was significantly different from 0, 1.0 (95% CI: 0.5–1.6, *P* < 0.0001). The incidence rate was lowest in 1974, 1975, 1979, 1991, and 1997 and highest in 1976, 1992, 1998–2000, 2002, 2003, and 2007. Remarkably, the rate ratio in 2007 (RR: 1.34 per 100000, 95% CI: 1.01–1.75), implying 34% excess thyroid cancer, was significantly different from the rate in 1973–2007, *P* < .0001.

Table 2 shows the incidence rate of pediatric thyroid cancer by race in 1973–2007 in the SEER registries. The incidence rate for white children ranged from 0.4 to 0.8 per 100,000. Among white children, the incidence was lowest in 1975, 1981, and 1997 and highest in 2001–2003 and 2007. However, none of these rates were statistically significantly different from the rate in 1973–2007 (*P* > 0.05). Relative to white children, the incidence rate was lower in blacks (Figure 2), ranging from 0.0 to 0.4 per 100000. In contrast, several RRs in 2001–2007 were statistically significantly lower compared to 1973–2007 rates in blacks.

**Figure 1:**
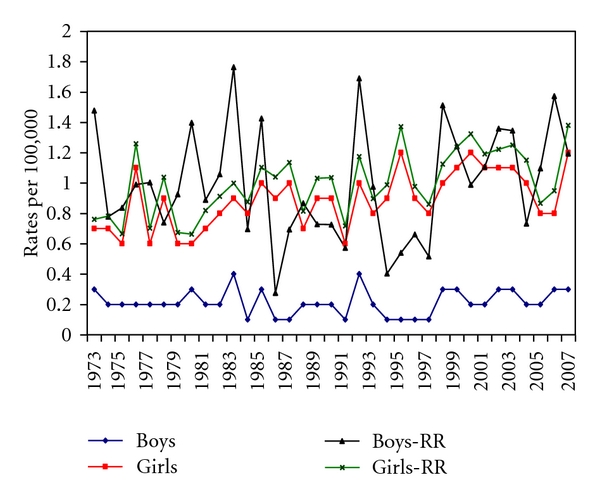
Age-adjusted incidence rate and rate ratio of pediatric thyroid carcinoma by sex, SEER dataset, in 1973–2007.

**Figure 2:**
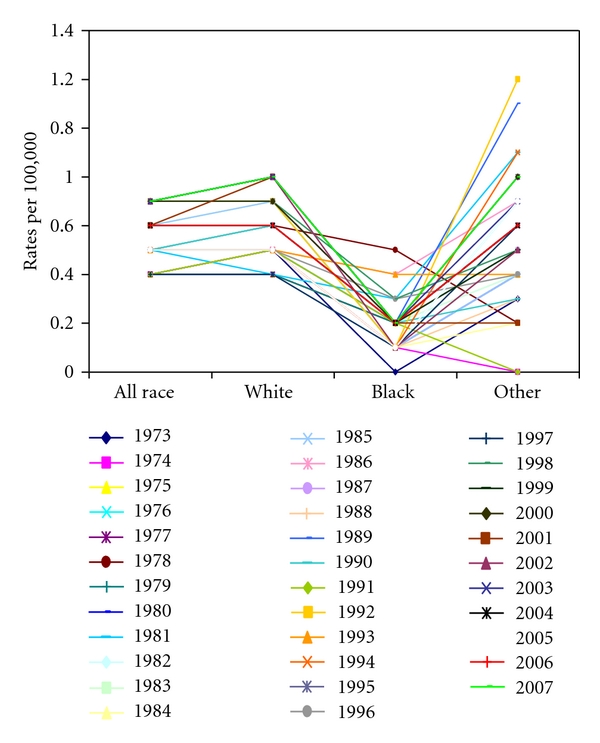
Age-adjusted incidence rate of pediatric thyroid carcinoma by race, SEER dataset, in 1973–2007.

Table 3 illustrates the RR of pediatric thyroid cancers by sex in the SEER registries in 1973–2007. Overall, the rate was slightly higher among girls. Among boys, the RR ranged from 0.4 to 1.8 per 100000, with the highest RR observed in 1983 (RR: 1.8, 95% CI: 0.93–3.07) and the lowest in 1994 (RR: 0.4, 95% CI: 0.08–1.18). Among girls, the RR ranged from 0.72 to 1.38 per 100,000, with the highest RR observed in 2007 (RR: 1.38, 95% CI: 1.01–1.85). Whereas in girls, the RR in 2007 was statistically significantly different from the rate in 1973–2007, that was not the case among boys. There were fewer thyroid cancers diagnosed among boys (267 cases, *n* = 126,581,849, rate (*R*): 0.2 per 100,000), compared to girls (1, 093 cases, *n* = 121,056,935, *R*: 0.9 per 100,000). But overall, there was a significant increase in thyroid cancer in 2007 (*R*: 0.7 per 100,000, RR: 1.34, 95% CI: 1.01–1.75) relative to 1973–2007 (*R*: 0.5 per 100,000, 95% CI: 0.6–0.9). Similarly, among girls, there was a significant increase in thyroid cancer in 2007 (Figure 1, *R*: 1.2 per 100,000, RR: 1.38, 95% CI: 1.01–1.85, *P* = 0.04). While at any given year, the trend was higher among girls relative to boys, there was a negative trend in boys (PC: −19.2, nonsignificant APC: 0.4), while a positive significant trend was observed among girls (PC: 81.3, APC: 1.2, 95% CI: 0.6–1.8, *P* < 0.0001).

Table 4 demonstrates age-specific incidence rate for pediatric thyroid carcinoma by age group at diagnosis in the SEER registries in 1973–2007. Among children of age 0–4 years, the rate ranged from 0.0 to 0.1 with four cancers diagnosed between 1973 and 2007, while among the age group of 5–9 years the rate ranged from 0.0 to 0.3 per 100,000. In the age group of 10–14 years thyroid carcinoma rate was 0.2–0.9 with the rate in 2002 (R: 0.9) statistically significantly different from 1973–2007. Among children in age group of 15–19 years, thyroid cancer rate ranged from 1.0 to 2.3 per 100000, with the rate in 1975 significantly different from 1973–2007. There was no thyroid carcinoma diagnosed during the first 12 months of life (*n* = 12,495,777); in the age group of 1–4 years (*n* = 48,601,741), there were 4 cases of thyroid cancer (1976, 1986, 1993, and 2007), with the rate of 0.1 per 100,000. In the age group of 5–9 years (*n* = 60,757,527), there were 54 tumors diagnosed, while, in the age group of 10–14 years (*n* = 62,502,949), 281 carcinomas were diagnosed, with the rate in 1992 significantly different from the rate between 1973 and 2007 (rate: 0.9 per 100,000, RR: 1.93, 95% CI: 1.06–3.23, *P* = 0.03). Data were insufficient to demonstrate trends in age group of 0–4 years, but, among 5–9, there was a negative but insignificant trend (PC: −62.0), while positive trends were shown among the age groups of 10–14 years (PC: 55.0) and 15–19 years (PC: 53.4). While the APCs could not be computed for ages 5–9 years, the APC was insignificantly higher than zero in the age group of 10–14 years but was significantly higher than 0 in the age group of 15–19 years (APC: 1.1, 95% CI: 0.5–1.8, *P* < 0.0001). Among the age group of 15–19 years (*n* = 63,280,790), there were 1021 tumors diagnosed, with rates fluctuating between 1.0 and 2.3 per 100,000.

Table 5 illustrates the age-adjusted incidence rate of pediatric thyroid cancer by the 9 SEER registries in 1973–2007. In SA the rate ranges from 0.1 to 1.3, and in Connecticut the rate was between 0.1 and 1.3 as well, whereas in Detroit MP, the rate was 0.2 (1980), and 0.8 in 1982 and 1983. There were positive though insignificant trends in Utah (PC: 119.3), New Mexico (PC: 312), and Iowa (PC: 108.7, APC 0.8), but significant in Connecticut (PC: 312.0, APC: 2.1, *P* < 0.0001). In contrast, nonsignificant negative trends were observed in Detroit metropolitan (PC: −20.7), Hawaii (PC: −16.8), and San Francisco-Oakland (PC: −47.7, APC: 0.3, *P* > 0.05).

## 4. Discussion 

This study was conducted to assess whether the incidence of pediatric carcinoma has been increasing in the United States during the past three decades. We examined the incidence rate and trends over this period by sex, age at diagnosis, race, and geographic locale. This investigation revealed a few relevant findings: first there is a fluctuating pattern on age-adjusted incidence rate in pediatric thyroid cancer based on the nine SEER registries utilized in this study. Second, pediatric thyroid carcinoma incidence varies by age at diagnosis, race, and sex. Third, there is a clear pattern in the age at diagnosis, with incidence increasing as the age increases. 

Pediatric thyroid carcinoma is a rare malignancy and has a good prognosis except the anaplastic cell type. The papillary, follicular, and medullary subtypes, which are collectively termed well-differentiated tumors, have very good prognosis [15]. We have shown increase in the pattern of thyroid cancer diagnosed among pediatric patients (0–19 years) in the USA between 1973 and 2007. Despite this increase, we observed a fluctuating pattern with most increases seen in the new millennium. Of the risk factors investigated in thyroid cancer epidemiology, ionizing radiation remains a very well-established finding with a perpetual consistency across study populations and types of designs [13, 14]. The observed increased rate of pediatric thyroid cancer in our study may be associated with similar increase in other pediatric malignancies due to the increasing use of radiation therapy in the management of these tumors in our pediatric populations. Relative to adults, the thyroid gland in children is most sensitive to ionizing radiation, [13] which explains the increasing proportion of thyroid cancer diagnosed in older children who underwent radiation therapy for their first primary tumor [16]. Our study showed that the incidence rate ranged from 0.4–0.7 per 100000 in the USA. Whereas there are no long-term temporal trend studies to our knowledge, the limited studies done in these perspectives identified incidence of pediatric thyroid cancer to be one in every 1000–2000 children [17].

We have also demonstrated that the incidence rate varied by race and that white children compared to black children had higher incidence of thyroid cancer. While the increasing incidence trend was not significant among white children, the lower trends among black children were statistically significant between 1973–2007. The observed variability by race in pediatric thyroid cancer remains to be explained by examining factors known to drive thyroid cancer that are disproportionately distributed between black and white children in the US population. Whereas this study did not assess variability by race in the factors predisposing to thyroid cancer, it is plausible to expect that the observed race variability in the incidence of thyroid cancer in our data may be associated with racial variability in first primary cancer therapeutics (radiation therapy), mortality from primary cancer, secondary exposure to tobacco, diet, and parental occupation. 

Thyroid cancer incidence was observed to vary by sex, with girls exhibiting increased incidence relative to boys. This observation, which supports previous data in young adult and adult populations (14 years and older) [3, 6, 7], may be explained in part by hormonal differences by sex. Estrogen receptors in normal and neoplastic human thyroid tissues had been investigated by Chaudhuri and Prinz [5]. Estrogen may be associated with thyroid cancer since it is related with the elevation of serum thyroxin and tri-iodothyronine levels [18]. The fact that the variability exits in thyroid cancer incidence by sex due to hormonal differences is clearly supported by data on age at diagnosis of thyroid cancer. Since this malignancy peaks during puberty, increasing in this pattern until middle age [2, 7]. In addition, thyroid-stimulating hormone (TSH) from the anterior pituitary gland may be carcinogenic by increasing mitotic activity in the follicular cell. Consequently, TSH elevation may be implicated in thyroid carcinoma carcinogenesis [19]. 

We found that pediatric thyroid cancer rate varied by age at diagnosis and that the incidence was highest among children of 15–19 years and negligible among children less than one year with no incidence between 1973 to 2007. Ferlay et al. observed a similar pattern in adult thyroid cancer population [20]. Incidence of thyroid cancer has been shown to be relatively high before age 40 years, and in our data with the pediatric population we observed highest incidence rate among children of 15–19 years, which may be suggestive of hormonal role in thyroid carcinogenesis. Equally this implication helps to explain the sex variability in pediatric thyroid cancer rate. The fact that we observed increasing rate from age group of 10–19 years is also suggestive of the implication of hormones including oral contraceptives (15–19 years) in girls and age at onset variability in pediatric thyroid cancer in both sexes. 

Whereas the role of the environment has not been consistently implicated in thyroid cancer carcinogenesis, certain environment including physical environment or geography and medical environment (diagnostics and therapeutics, occupation) may influence thyroid cancer incidence. We studied temporal trend in the nine SEER registries which began data collection in 1973 except Seattle and Atlanta Metropolitan. While no specific patterns were observed, registries with white pediatric population tended to show slight increase in incidence rates. This observation is suggestive of the role of race as a surrogate environmental predisposing factor in pediatric thyroid malignancy. 

While this study represents the largest and the longest temporal trend study in pediatric thyroid carcinoma in North America, there are some limitations. First, the population studied represented an estimated 11% of the total US population, which may render our result an underestimation or overestimation of the incidence rate in pediatric thyroid carcinoma. However, the observed incidence rate in general and the incidence rate variability by sex, race, and geography do not appear to be overly an underestimation. We could have included other SEER registries that could have made our data representative of an estimated 26% of the US population. But doing so, we could have reduced the number of years of tumor diagnosis since some of these current registries started data collection in the late 90s and early 2000s. 

In summary, this temporal trend study of pediatric thyroid carcinoma indicates an increase in the incidence pattern of this malignancy given the percent change and the annual percent change between 1973 and 2007. In addition, the incidence is higher among girls, lower among blacks, and relatively higher in SEER registries with predominantly white or Hispanic populations. Since changes in diagnostics, and population transition, and other limitations of disease registry such as SEERS may substantially influence incidence patterns, rates, and trends, caution is expected in the interpretation of these results.

## Figures and Tables

**Table 1:** Pediatric thyroid cancer incidence by year of diagnosis, SEER dataset, in 1973–2007.

Year of diagnosis	Population at risk	Tumor count	Rate	RR	95% CI	
					LL	UL
1973–2007	247,638,784	1,360				

1973	5,982,303	31	0.5	0.91	0.61	1.30
1974	6,706,606	31	0.4	0.78	0.53	1.12
1975	7,182,481	30	0.4	0.70	0.47	1.01
1976	7,094,067	51	0.7	1.21	0.90	1.61
1977	7,005,266	32	0.4	0.76	0.52	1.09
1978	6,930,226	41	0.5	0.98	0.70	1.35
1979	6,867,527	30	0.4	0.73	0.49	1.05
1980	6,825,778	33	0.4	0.81	0.55	1.15
1981	6,768,320	33	0.5	0.83	0.57	1.18
1982	6,699,252	36	0.5	0.94	0.66	1.31
1983	6,652,577	43	0.6	1.15	0.83	1.56
1984	6,604,197	31	0.5	0.84	0.57	1.20
1985	6,595,047	42	0.6	1.17	0.84	1.59
1986	6,591,444	32	0.5	0.89	0.61	1.26
1987	6,611,620	38	0.6	1.05	0.74	1.45
1988	6,654,692	29	0.4	0.82	0.55	1.19
1989	6,705,175	34	0.5	0.97	0.67	1.36
1990	6,789,031	34	0.5	0.97	0.67	1.37
1991	6,856,321	24	0.4	0.69	0.44	1.02
1992	6,945,467	45	0.7	1.27	0.92	1.71
1993	7,063,402	33	0.5	0.91	0.62	1.28
1994	7,159,479	32	0.5	0.87	0.59	1.23
1995	7,251,171	45	0.7	1.20	0.87	1.62
1996	7,344,331	35	0.5	0.91	0.63	1.27
1997	7,425,322	31	0.4	0.79	0.53	1.12
1998	7,505,219	48	0.7	1.20	0.88	1.60
1999	7,562,024	50	0.7	1.23	0.91	1.64
2000	7,597,829	51	0.7	1.25	0.93	1.66
2001	7,623,698	48	0.6	1.17	0.86	1.56
2002	7,622,505	51	0.7	1.24	0.92	1.65
2003	7,627,054	52	0.7	1.27	0.94	1.67
2004	7,650,359	44	0.6	1.06	0.77	1.44
2005	7,665,955	38	0.5	0.91	0.64	1.26
2006	7,714,191	45	0.6	1.07	0.78	1.44
2007	7,758,848	57	0.7	1.34	1.01	1.75

LL: lower limit of confidence limit; UL: upper limit of confidence limit; CI: confidence interval; RR: rate ratio compared to 1973–2007.

**Table 2:** Incidence of pediatric thyroid cancer in the USA by race, SEER dataset, in 1973–2007.

Year of diagnosis	Whites			Blacks			Others		
	Rates	95% CI		Rates	95% CI		Rates	95% CI	
		LL	UL		LL	UL		LL	UL
1973	0.5	0.4	0.8	0	0	0.6	0.3	0	1.5
1974	0.5	0.3	0.7	0.1	0	0.9	0	0	0.9
1975	0.4	0.2	0.6	0.2	0	0.9	0.4	0.1	1.6
1976	0.7	0.5	0.9	0.1	0	0.7	0.7	0.1	1.9
1977	0.4	0.3	0.6	0.2	0	0.9	0.6	0.1	1.9
1978	0.6	0.4	0.8	0.5	0.1	1.2	0.2	0	1.1
1979	0.4	0.3	0.6	0.2	0	0.9	0.6	0.1	1.8
1980	0.5	0.3	0.7	0.1	0	0.7	0.4	0	1.4
1981	0.4	0.3	0.6	0.3	0.1	1	0.9	0.3	2.2
1982	0.6	0.4	0.8	0.2	0	0.9	0.4	0	1.3
1983	0.7	0.5	1	0.1	0	0.7	0.5	0.1	1.6
1984	0.5	0.4	0.8	0.1	0	0.7	0.2	0	1
1985	0.7	0.5	1	0.1	0	0.7	0.4	0	1.2
1986	0.5	0.3	0.7	0.4	0.1	1	0.7	0.2	1.8
1987	0.6	0.4	0.9	0.2	0	0.8	0.5	0.1	1.5
1988	0.5	0.3	0.8	0.1	0	0.6	0.3	0	1.2
1989	0.5	0.3	0.7	0.2	0	0.8	1.1	0.4	2.3
1990	0.6	0.4	0.9	0.2	0	0.8	0.3	0	1.1
1991	0.5	0.3	0.7	0.2	0	0.8	0	0	0.5
1992	0.7	0.5	1	0.1	0	0.6	1.2	0.5	2.4
1993	0.5	0.3	0.8	0.4	0.1	1.1	0.4	0.1	1.3
1994	0.5	0.3	0.7	0.1	0	0.6	0.9	0.3	1.8
1995	0.7	0.5	1	0.2	0	0.8	0.8	0.3	1.8
1996	0.5	0.3	0.8	0.3	0.1	0.9	0.4	0.1	1.1
1997	0.4	0.3	0.7	0.1	0	0.5	0.6	0.2	1.5
1998	0.7	0.5	1	0.3	0.1	0.9	0.5	0.1	1.2
1999	0.8	0.6	1	0.2	0	0.7	0.5	0.1	1.2
2000	0.7	0.5	1	0.2	0	0.7	0.8	0.3	1.7
2001	0.8	0.5	1	0.2	0	0.6	0.2	0	0.8
2002	0.8	0.6	1.1	0.1	0	0.5	0.5	0.1	1.2
2003	0.8	0.6	1.1	0.2	0	0.6	0.7	0.3	1.5
2004	0.6	0.4	0.9	0.2	0	0.6	0.6	0.2	1.3
2005	0.5	0.4	0.8	0.1	0	0.5	0.7	0.2	1.5
2006	0.6	0.4	0.9	0.2	0	0.6	0.6	0.2	1.3
2007	0.8	0.6	1.1	0.2	0	0.6	0.8	0.3	1.6

LL: lower limit of confidence limit; UL: upper limit of confidence limit; CI: confidence interval.

**Table 3:** Incidence of pediatric thyroid cancer in the USA by sex, SEER dataset, in 1973–2007.

Year of diagnosis	Males			Females		
	RR	95% CI		RR	95% CI	
		LL	UL		LL	UL
1973	1.48	0.70	2.79	0.76	0.47	1.18
1974	0.78	0.28	1.75	0.78	0.50	1.17
1975	0.84	0.33	1.78	0.67	0.42	1.01
1976	0.99	0.42	2.01	1.26	0.90	1.71
1977	1.00	0.43	2.03	0.70	0.45	1.06
1978	0.74	0.27	1.66	1.04	0.72	1.46
1979	0.93	0.37	1.95	0.67	0.43	1.02
1980	1.40	0.69	2.56	0.66	0.41	1.01
1981	0.89	0.35	1.88	0.82	0.53	1.21
1982	1.06	0.45	2.12	0.91	0.60	1.33
1983	1.76	0.93	3.07	1.00	0.67	1.44
1984	0.69	0.22	1.64	0.88	0.57	1.29
1985	1.43	0.68	2.66	1.10	0.75	1.57
1986	0.28	0.03	1.01	1.04	0.70	1.49
1987	0.69	0.22	1.64	1.14	0.78	1.61
1988	0.87	0.32	1.90	0.81	0.51	1.23
1989	0.73	0.23	1.70	1.03	0.69	1.49
1990	0.73	0.23	1.69	1.04	0.69	1.49
1991	0.57	0.15	1.46	0.72	0.44	1.11
1992	1.69	0.86	2.99	1.17	0.80	1.66
1993	0.98	0.39	2.02	0.90	0.58	1.32
1994	0.40	0.08	1.18	0.99	0.66	1.43
1995	0.54	0.15	1.38	1.37	0.98	1.87
1996	0.66	0.21	1.55	0.98	0.66	1.40
1997	0.51	0.14	1.33	0.86	0.56	1.26
1998	1.51	0.77	2.68	1.12	0.78	1.57
1999	1.24	0.59	2.32	1.24	0.88	1.70
2000	0.99	0.42	1.97	1.32	0.95	1.80
2001	1.11	0.50	2.13	1.19	0.84	1.64
2002	1.36	0.67	2.47	1.22	0.87	1.67
2003	1.35	0.66	2.44	1.25	0.89	1.71
2004	0.73	0.27	1.61	1.15	0.81	1.59
2005	1.10	0.50	2.11	0.87	0.58	1.25
2006	1.57	0.83	2.74	0.95	0.65	1.35
2007	1.19	0.57	2.23	1.38	1.01	1.85

LL: lower limit of confidence limit; UL: upper limit of confidence limit; CI: confidence interval; RR: rate ratio.

**Table 4:** Age-specific incidence of pediatric thyroid cancer in the USA, SEER dataset, in 1973–2007.

Year of diagnosis	0–4 years			5–9 years			10–14 years			15–19 years		
	Population	TC	Rate	Population	TC	Rate	Population	TC	Rate	Population	TC	Rate
1973	1,072,245	0	0	1,432,428	2	0.1	1,646,082	5	0.3	1,584,706	24	1.5
1974	1,176,199	0	0	1,579,384	0	0	1,851,090	7	0.4	1,827,895	24	1.3
1975	1,229,920	0	0	1,688,020	0	0	1,971,683	10	0.5	1,991,191	20	1.0
1976	1,188,688	1	0.1	1,686,887	3	0.2	1,913,149	12	0.6	2,006,411	35	1.7
1977	1,171,997	0	0	1,665,895	3	0.2	1,853,142	5	0.3	1,999,246	24	1.2
1978	1,187,830	0	0	1,637,991	4	0.2	1,794,352	4	0.2	1,988,727	33	1.7
1979	1,214,570	0	0	1,599,133	2	0.1	1,746,549	4	0.2	1,974,474	24	1.2
1980	1,243,643	0	0	1,564,337	2	0.1	1,723,562	4	0.2	1,946,521	27	1.4
1981	1,284,334	0	0	1,519,291	3	0.2	1,723,711	3	0.2	1,887,046	27	1.4
1982	1,308,683	0	0	1,513,782	2	0.1	1,697,129	9	0.5	1,823,596	25	1.4
1983	1,335,696	0	0	1,532,229	0	0	1,664,109	13	0.8	1,764,760	30	1.7
1984	1,352,298	0	0	1,566,143	0	0	1,618,140	5	0.3	1,721,192	26	1.5
1985	1,352,569	0	0	1,605,425	2	0.1	1,576,537	10	0.6	1,705,295	30	1.8
1986	1,357,223	1	0.1	1,644,105	1	0.1	1,524,050	7	0.5	1,710,362	23	1.3
1987	1,365,655	0	0	1,672,003	1	0.1	1,520,948	4	0.3	1,696,923	33	1.9
1988	1,376,921	0	0	1,697,507	1	0.1	1,541,516	9	0.6	1,676,400	19	1.1
1989	1,401,082	0	0	1,709,902	0	0	1,579,061	9	0.6	1,641,653	25	1.5
1990	1,430,960	0	0	1,726,047	0	0	1,631,541	3	0.2	1,612,817	31	1.9
1991	1,463,423	0	0	1,742,886	2	0.1	1,686,781	6	0.4	1,579,712	16	1
1992	1,491,441	0	0	1,752,403	1	0.1	1,731,748	15	0.9	1,589,984	29	1.8
1993	1,516,540	1	0.1	1,774,688	4	0.2	1,779,296	7	0.4	1,619,730	21	1.3
1994	1,519,630	0	0	1,808,126	2	0.1	1,800,486	8	0.4	1,663,588	22	1.3
1995	1,505,360	0	0	1,848,547	0	0	1,816,398	7	0.4	1,716,488	38	2.2
1996	1,491,423	0	0	1,888,341	0	0	1,834,425	4	0.2	1,769,159	31	1.8
1997	1,477,356	0	0	1,925,871	0	0	1,847,037	10	0.5	1,811,580	21	1.2
1998	1,473,425	0	0	1,951,753	3	0.2	1,867,304	7	0.4	1,847,826	38	2.1
1999	1,472,530	0	0	1,959,332	5	0.3	1,902,079	7	0.4	1,860,877	38	2
2000	1,471,882	0	0	1,945,304	3	0.2	1,937,644	10	0.5	1,869,474	38	2
2001	1,472,853	0	0	1,919,747	2	0.1	1,967,159	14	0.7	1,873,398	32	1.7
2002	1,489,013	0	0	1,890,526	0	0	1,984,118	12	0.6	1,873,406	39	2.1
2003	1,503,563	0	0	1,864,486	0	0	1,989,481	12	0.6	1,879,583	40	2.1
2004	1,524,279	0	0	1,850,825	2	0.1	1,982,348	11	0.6	1,898,838	31	1.6
2005	1,546,678	0	0	1,843,731	1	0.1	1,955,089	9	0.5	1,929,234	28	1.5
2006	1,556,428	0	0	1,865,235	2	0.1	1,933,272	10	0.5	1,959,038	33	1.7
2007	1,575,404	1	0.1	1,885,217	1	0.1	1,911,933	9	0.5	1,979,660	46	2.3

TC: tumor count. The population represents the pediatric population of 0–19 years in the SEER registries.

**Table 5:** Incidence of pediatric thyroid cancer in the USA by SEER registries in 1973–2007.

Year of diagnosis	San Francisco-Oakland SMSA	CT	Detroit (Metropolitan)	Hawaii	Iowa	NM	Seattle (Puget Sound)	Utah	Atlanta (Metropolitan)
1973	0.7	0.2	0.5	1.5	0.3	0.2	—	0.6	—
1974	0.4	0.4	0.4	0	0.1	1.2	0.7	0.6	—
1975	0.5	0.2	0.3	0.9	0.4	0.2	0.6	0.6	0.3
1976	0.5	1.1	0.6	0.6	0.8	0.6	0.4	1	0.2
1977	0.5	0.4	0.3	0.6	0.3	0.4	0.5	0.4	0.7
1978	0.4	0.6	0.5	0.3	0.9	1.2	0.6	0.2	0.2
1979	0.5	0.5	0.4	0.9	0.2	0.6	0.4	0.4	0
1980	0.6	0.4	0.2	0.6	0.5	0.4	0.3	0.7	0.5
1981	0.7	0.4	0.6	1.2	0.4	0.2	0.3	0.2	0.2
1982	0.7	0.1	0.8	0.6	0.8	0.0	0.5	0.3	0.3
1983	1	0.4	0.8	0.7	0.5	0.8	0.6	0.8	0
1984	0.4	0.4	0.6	0.3	0.8	0.4	0.2	0.6	0.2
1985	0.4	0.5	0.6	0.3	0.7	1.7	0.6	0.5	0.5
1986	0.6	0.5	0.6	0.7	0.4	0.2	0.5	0.5	0.3
1987	0.4	0.3	0.6	0.4	1	0.4	0.7	0.7	0.5
1988	0.5	0.3	0.6	0.3	0.6	0.4	0.1	0.7	0.3
1989	0.8	0.5	0.3	1	0.4	0.2	0.7	0.8	0.3
1990	0.9	0.6	0.5	0.3	0.6	1.1	0.2	0.3	0.2
1991	0.2	0.5	0.4	0.7	0.1	0.2	0.3	0.4	0.7
1992	1.3	1.1	0.6	1	0.7	0.2	0.4	0.0	0.8
1993	0.6	0.5	0.5	0.6	0.6	0.8	0.3	0.5	0.2
1994	0.8	0.2	0.6	0.6	0.2	0.2	0.6	0.1	0.8
1995	0.6	0.1	0.6	0.6	1.1	0.7	0.6	1	0.6
1996	0.6	0.6	0.5	0.9	0.6	0.4	0.4	0.4	0.4
1997	0.4	0.1	0.5	1.2	0.3	0.4	0.4	0.6	0.4
1998	0.1	0.6	0.7	0.9	0.8	0.5	0.9	0.8	0.5
1999	0.7	1.3	0.5	0.3	0.9	0.5	0.7	0.5	0.4
2000	0.8	0.9	0.6	0.6	0.6	0.7	0.8	0.8	0.3
2001	0.6	1.2	0.5	0.3	0.7	0.7	0.5	0.6	0.4
2002	0.6	0.5	0.6	0.3	0.9	0.9	0.8	0.4	1
2003	0.6	0.9	0.5	0.3	0.8	0.9	0.7	0.7	0.6
2004	0.5	0.8	0.3	0.6	0.5	1.1	0.4	0.6	0.6
2005	0.3	0.7	0.4	1.2	0.4	0.5	0.8	0.4	0.2
2006	0.8	0.8	0.6	0	0.4	0.9	0.8	0.1	0.4
2007	0.4	0.7	0.4	1.3	0.6	0.9	0.8	1.3	0.7
